# Patterns of the Nutrients and Metabolites in *Apostichopus japonicus* Fermented by *Bacillus natto* and Their Ability to Alleviate Acute Alcohol Intoxication

**DOI:** 10.3390/foods13020262

**Published:** 2024-01-14

**Authors:** Xingyu Gu, Ran Zhao, Haiman Li, Xinyu Dong, Meishan Meng, Tingting Li, Qiancheng Zhao, Ying Li

**Affiliations:** 1 College of Food Science and Engineering, Dalian Ocean University, Dalian 116023, China; guxingyu2022@163.com (X.G.); zhaoran2626@163.com (R.Z.); 15942109475@163.com (H.L.); 19818925753@163.com (X.D.); 15041435403@163.com (M.M.); 2 Liaoning Provincial Marine Healthy Food Engineering Research Centre, Dalian 116023, China; 3 Dalian Key Laboratory of Marine Bioactive Substances Development and High Value Utilization, Dalian 116023, China; 4 Key Laboratory of Biotechnology and Bioresources Utilization, Dalian Minzu University, Dalian 116650, China; jwltt@dlnu.edu.cn; 5 Collaborative Innovation Center of Seafood Deep Processing, Dalian Polytechnic University, Dalian 116034, China

**Keywords:** *Apostichopus japonicus*, *Bacillus natto*, fermentation, metabolome, acute alcohol intoxication

## Abstract

The aim of this study was to understand the changes in nutrient composition and differences in metabolites in *Apostichopus japonicus* fermented by *Bacillus natto* and their function in alleviating acute alcohol intoxication (AAI) through in vivo studies. The results showed no significant difference between the basic components of sea cucumber (SC) and fermented sea cucumber (FSC). The SC proteins were degraded after fermentation, and the amino acid content in FSC was significantly increased. The differentially abundant metabolites of SC and FSC were identified by LC-MS/MS. The contents of amino acid metabolites increased after fermentation, and arachidonic acid metabolism was promoted. The results demonstrated that FSC alleviated AAI by improving the activities of alcohol-metabolizing enzymes and antioxidant enzymes in the liver but did not alleviate the accumulation of triglycerides. Our results will provide beneficial information for the development and application of new products from FSC.

## 1. Introduction

Acute alcohol intoxication (AAI) occurs after a large amount of alcohol is ingested in a short period of time, and its incidence from family gatherings, socializing, and emotional venting is increasing [1]. The clinical manifestations of AAI mainly include behavioral and neurological symptoms accompanied by organ damage, and in severe cases, autonomic dysfunction, respiratory depression, coma, and cardiac arrest can result [2]. Ethanol dehydrogenase and acetaldehyde dehydrogenase are crucial enzymes during AAI, as they promote the conversion of ethanol to acetic acid. Subsequently, acetic acid is thoroughly decomposed into CO_2_ and H_2_O through the tricarboxylic acid cycle [3]. Currently, many studies have shown that probiotics have a positive effect on AAI. Lu et al. [4] successfully constructed a food-grade recombinant *Bacillus subtilis* strain via double-crossover homologous recombination, and studies have shown that this recombinant *B. subtilis* can alleviate AAI and alcohol-induced liver injury. Chen et al. [5] investigated the ability of ethanol-induced Lactococcus lactis intracellular extracts to alleviate alcoholism in mice, and the results showed that the extracts could effectively reduce the blood alcohol content. Moreover, the biochemical indices indicated that the extracts protected the liver and improved alcohol metabolism in the mice. However, the changes in nutrient composition and the potential mechanisms by which fermentation by probiotics can alleviate AAI have not yet been completely elucidated.

The sea cucumber *Apostichopus japonicus*, an important representative commercial marine species, has attracted much attention due to its rich nutritional composition and medicinal value [6,7,8,9]. These characteristics have led to an increasing demand for sea cucumber, which has further promoted the development of the sea cucumber processing industry. Various sea cucumber treatment and processing methods have been expanded upon, such as those for the development of instant sea cucumbers, dried sea cucumbers, and deep-processed sea cucumbers [6,10]. The main method by which sea cucumbers are deep processed is by degrading sea cucumber protein into sea cucumber polypeptide [11], which is more easily absorbed by the human body [12]. In addition, fermentation provides new insights into the degradation of sea cucumber protein. Studies have found that sea cucumber protein is effectively degraded by *Bacillus natto* fermentation, after which the amino acid content is significantly increased [13]. However, fermentation by microorganisms is accompanied by a complex material transformation. In addition to the degradation of sea cucumber protein, how the nutrient composition and function of sea cucumber change directly affects the application of fermentation as a method for the deep processing of sea cucumber [14].

In a previous study, we discussed the structure, in vitro digestion characteristics, and effect on the gut microbiota of sea cucumber polysaccharides fermented by *Bacillus natto* [14]. The aim of this study was to examine the nutritional composition and metabolites of sea cucumber before and after fermentation by nontargeted metabolomics. Based on a mouse model of AAI, the effect and potential mechanism of sea cucumber before and after fermentation on AAI were investigated. This study provides new insights into the development of new sea cucumber fermentation products and the prevention of AAI.

## 2. Results

### 2.1. Analysis of the Basic Compositions of FSC and SC

Nutritional properties before and after fermentation have a significant impact on physiological activity. The basic composition analyses of FSC and SC are shown in Table 1. The protein contents of SC and FSC were 52.97 ± 1.02% and 56.28 ± 1.12%, the total sugar contents were 9.17 ± 0.87% and 10.32 ± 0.56%, the fat contents were 2.50 ± 0.12% and 2.60 ± 0.24%, the ash contents were 17.38 ± 1.04% and 18.05 ± 0.85%, and the moisture contents were 3.97 ± 0.25% and 4.61 ± 0.23%, respectively. These results showed that there were no significant differences in the basic compositions of SC and FSC.

The essential amino acid contents in SC and FSC were 10.52 g/100 g and 11.84 g/100 g, respectively, and the nonessential amino acid contents were 22.24 g/100 g and 27.61 g/100 g, respectively. After SC fermentation, both the essential and nonessential amino acid contents increased. It is worth noting that there was a significant increase in the nonessential amino acids arginine, glycine, tyrosine, and serine and a significant decrease in the content of histidine, and significant increases in the contents of the essential amino acids isoleucine, threonine, and leucine.

### 2.2. Protein Characterization

The contents of water-soluble proteins of SC and FSC were determined. The results showed that the water-soluble protein contents of SC and FSC were 3.60 ± 0.04 mg/mL and 11.20 ± 0.16 mg/mL, respectively. The soluble protein contents of FSC were significantly higher than those of SC, which indicated that the fermentation of *Bacillus natto* increased the soluble protein content of SC.

**Figure 1:**
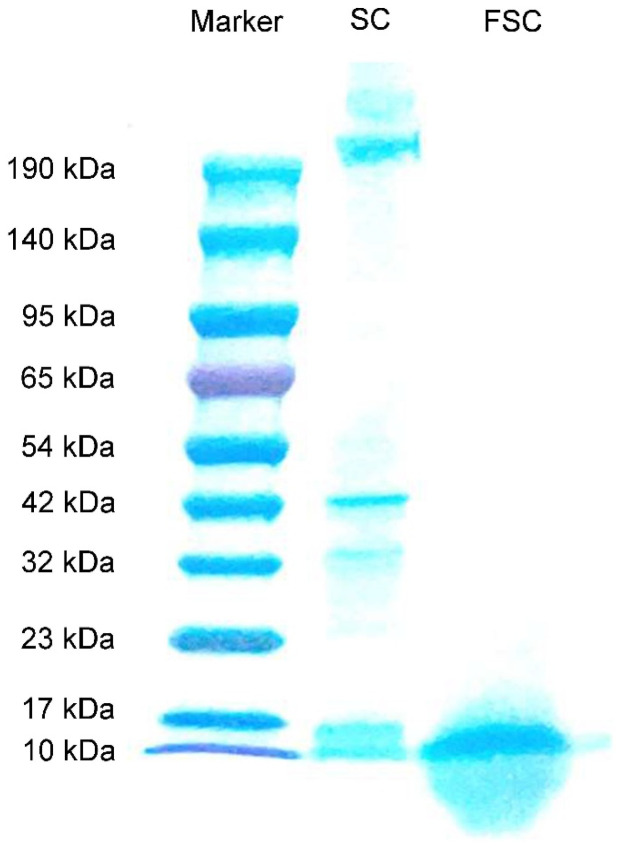
Tricine-SDS-PAGE analysis of SC and FSC. SC: sea cucumber; FSC: fermented sea cucumber.

The molecular mass distributions of FSC and SC determined by Tricine-SDS-PAGE are shown in Figure 1. The molecular mass distribution of SC proteins was distributed between 190 and 10 kDa, while FSC had obvious bands only near 17–10 kDa. This indicated that the protein in SC was degraded from large molecules to small molecules by fermentation, and *Bacillus natto* could effectively decompose the protein in SC.

### 2.3. Effects of FSC on AAI

#### 2.3.1. Blood Alcohol Content and Behavioral Analysis

The effects of FSC and SC on blood alcohol content and behavioral analyses of the treated animals were investigated, and the results are shown in Table 2. The higher the blood alcohol content, the more severe the alcohol intoxication symptoms. The blood alcohol content was not detected in the control group, while the blood alcohol content of the model group was 19.33 ± 2.22 μg/mL. The blood alcohol content in the FSC group was markedly lower than that in the model group, while there was no significant difference between the SC group and the model group. The results showed that gavage of FSC reduced the blood alcohol content of AAI mice.

The behavioral characteristics of the AAI mice were directly related to the blood alcohol content. After gavage, the mice in the control group drank and ate normally, while most of the remaining mice showed limpness and lethargy. In addition to the mice in the control group, the incidence of loss of the righting reflex was 100% in the other five groups, indicating that the AAI mouse model had been successfully established. The average latency time and duration of mice in the model group were 13.11 ± 3.85 min and 239.00 ± 74.56 min, respectively, and there was no significant difference in the average latency time and duration between the mice in the model group and the L-FSC group. However, the average latency time of the mice in the H-FSC group was significantly prolonged, and the average duration was significantly shortened. These results indicated that the incidence of AAI did not change in the H-FSC group, but these mice had an improved tolerance to AAI.

#### 2.3.2. Effect of FSC on Liver Injury

The liver index and triglyceride content of the mice were measured, and the results are shown in Table 3. There were no significant differences in the liver index between the groups, but the triglyceride content in the livers of the mice in the remaining groups was significantly elevated compared with that in the control group. The continuous intake of alcohol over a short period elevated the triglyceride content in the livers but did not cause the mice to suffer from fatty liver, and the intake of FSC did not alleviate the accumulation of triglycerides in the livers.

The plasma levels of aspartate aminotransferase and alanine aminotransferase of the mice were measured, and the results are shown in Table 3. Compared with the control group, the plasma alanine aminotransferase and aspartate aminotransferase contents were significantly increased in the model group, indicating that the continuous intake of high doses of alcohol in a short period caused hepatocellular injury in mice. In the positive group, the plasma alanine aminotransferase and aspartate aminotransferase contents were also significantly increased, indicating that RU21 did not alleviate hepatocellular injury in mice. In the SC and FSC groups, the plasma alanine aminotransferase and aspartate aminotransferase contents were significantly decreased to the level of the control group, suggesting that both SC and FSC could alleviate hepatocellular injury caused by the continuous intake of alcohol in the short term.

#### 2.3.3. Analysis of the Liver Tissue Biochemical Indices

**Figure 2:**
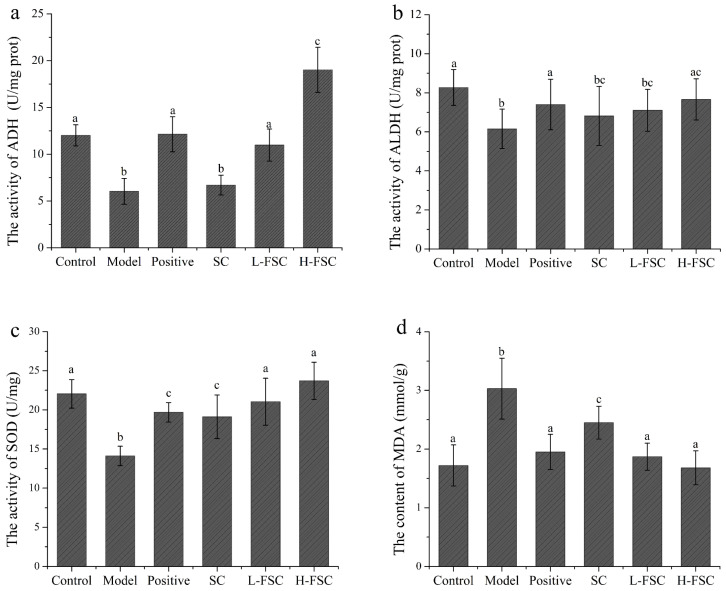
Effect of FSC on liver tissue biochemical indices. (**a**) Activity of ethanol dehydrogenase; (**b**) activity of acetaldehyde dehydrogenase; (**c**) activity of superoxide dismutase; (**d**) content of malondialdehyde. Control: control group; Model: model group; Positive: positive group; SC: SC group; L-FSC: low dose FSC group; and H-FSC: high dose FSC group. Data having different letters indicates that the difference is significant (*p <* 0.05).

To further investigate the mechanism by which FSC alleviates AAI in mice, the activities of ethanol dehydrogenase and acetaldehyde dehydrogenase in the livers were measured, and the results are shown in Figure 2a,b. Compared with the control group, the activities of ethanol dehydrogenase and acetaldehyde dehydrogenase in the model group were significantly reduced. The activities of ethanol dehydrogenase and acetaldehyde dehydrogenase in the positive group were increased to the levels in the control group, suggesting that RU21 exerted a protective effect against AAI in mice by activating ethanol dehydrogenase and acetaldehyde dehydrogenase and thus accelerating the catabolism of ethanol. The activities of ethanol dehydrogenase and acetaldehyde dehydrogenase in the SC group were also significantly reduced, indicating that SC had no effect on the activities of ethanol dehydrogenase and acetaldehyde dehydrogenase. It is worth noting that the activity of ethanol dehydrogenase in the L-FSC group was close to that in the positive group and ethanol dehydrogenase activity in the H-FSC group was more than 50% that in the positive group. The FSC group showed a significant increase in the activity of ethanol dehydrogenase. The acetaldehyde dehydrogenase activity in the L-FSC group was significantly lower than that in the control group, but acetaldehyde dehydrogenase activity in the H-FSC group was close to that in the control group. The above results indicated that FSC could activate ethanol dehydrogenase and acetaldehyde dehydrogenase.

The malondialdehyde content and superoxide dismutase activity in the livers were analyzed, and the results are shown in Figure 2c,d. The malondialdehyde content in the model group was significantly higher than that in the control group, while the malondialdehyde contents in the remaining four groups were reduced to different degrees. The malondialdehyde content in the H-FCS group was lower than that in the L-FSC group. The superoxide dismutase activity in the model group was significantly lower than that in the control group. Compared with the positive and SC groups, gavage of FSC produced a larger increase in superoxide dismutase activity in mice. Superoxide dismutase activity in the H-FSC group was higher than that in the L-FSC group. These results showed that both SC and FSC could significantly reduce the malondialdehyde content and increase the superoxide dismutase activity in the mouse livers, but the effect of FSC was more significant.

### 2.4. Metabolomics Analysis

#### 2.4.1. Screening Differentially Abundant Metabolites

**Figure 3:**
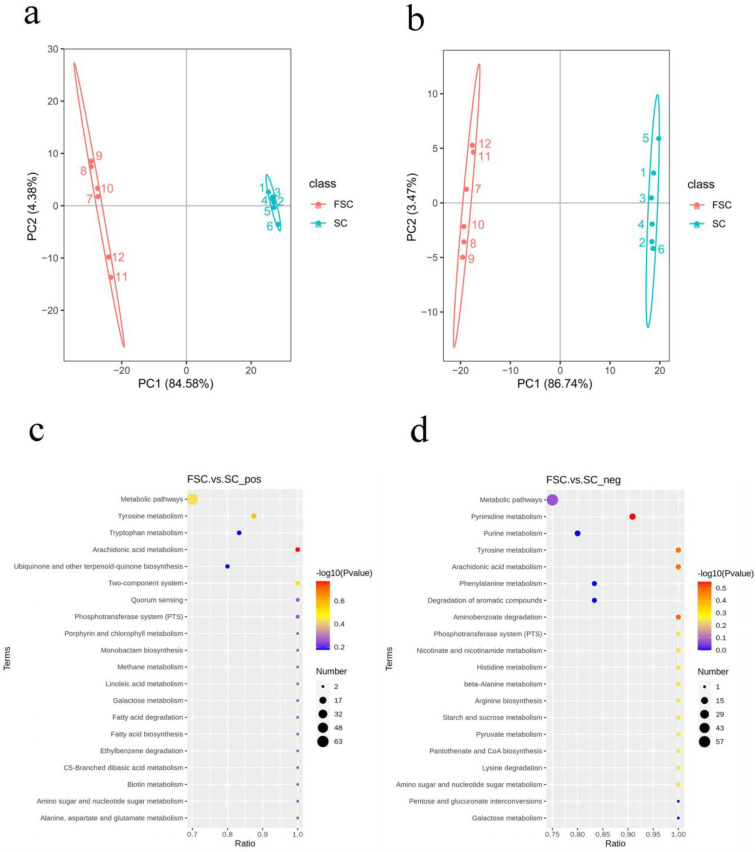
Principal component analysis and KEGG enrichment analysis of the SC and FSC metabolomes. Principal component analysis of the positive (**a**) and negative (**b**) ionization mode data. KEGG enrichment analysis of the data from positive (**c**) and negative (**d**) ionization mode. The linear distance between the two samples in (**a**,**b**) represents the difference in the metabolites. The horizontal axis in the figure is x/y (the number of differentially abundant metabolites in the corresponding metabolic pathway/the total number of metabolites identified in the pathway), and the higher the value is, the more differentially abundant metabolites in the pathway there are. The color of the dot indicates the *p* value from the hypergeometric test, and the smaller the value is, the greater the reliability of the test and statistical significance of the difference. The size of the dot represents the number of differentially abundant metabolites in the corresponding pathway, and the larger the dot is, the more differentially abundant metabolites there are in the pathway.

To explore the potential mechanism by which FSC alleviates AAI, the differences in FSC and SC metabolites were analyzed by nontargeted metabolomics. The principal component analysis plots (Figure 3a,b) show that the SC and FSC groups were significantly separated, which indicated a significant difference in the metabolite level. The samples from the same group clustered together, which indicated that the sample differences within each group were small.

In this experiment, metabolites that satisfied *VIP* > 1 and *p <* 0.05 were selected as metabolites with significantly different abundances. Univariate statistical analysis of the positive ionization mode data indicated that between the FSC and SC groups, there were 676 significantly different metabolites, of which 324 were upregulated and 352 were downregulated. In negative ionization mode, 346 significantly different metabolites were found, of which 206 were upregulated and 140 were downregulated.

#### 2.4.2. Differentially Abundant Metabolites Related to Amino Acid Metabolism

Further analysis of the differentially abundant metabolites between FSC and SC revealed that many from both positive and negative ionization modes were related to amino acid metabolism (Supplementary Files Tables S1 and S2), such as L-tyrosine, L-arginine succinate, L-threonine, and L-aspartic acid, and among them, there were more upregulated than downregulated metabolites. The contents of amino acids and their metabolites that play essential roles in human health, such as threonine, aspartic acid, tyrosine, and methionine, showed an upward trend, indicating that the levels of amino acids and their metabolites increased after SC fermentation, corroborating the observed increase in the amino acid content in FSC.

#### 2.4.3. Kyoto Encyclopedia of Genes and Genomes (KEGG) Enrichment Analysis

In this experiment, KEGG enrichment analysis was performed to understand the connection between differentially abundant metabolites and metabolic pathways, predict the related functions based on the metabolic pathways, and screen the dominant pathways in the changes in the function of FSC. As shown in Figure 3c,d, the main enriched metabolic pathway found in positive ionization mode was arachidonic acid metabolism. The main enriched metabolic pathways found in negative ionization mode were pyrimidine metabolism, arachidonic acid metabolism, tyrosine metabolism, and amino benzoic acid metabolism. The metabolite involved in arachidonic acid metabolism that was upregulated in positive ionization mode was prostaglandin E2. The metabolites involved in pyrimidine metabolism in negative ionization mode were mainly pseudouridine, deoxyuridine, uridine, malonic acid, methylmalonic acid, and cytidine diphosphate. The arachidonic acid metabolic pathway was enriched in both positive and negative ionization modes, suggesting that arachidonic acid metabolism changed during the fermentation of sea cucumbers.

## 3. Discussion

In this study, we fermented sea cucumber with *Bacillus natto* using the optimized conditions in our previous study [14]. There was no effect of sea cucumber fermented by *Bacillus natto* on the structure and digestion of sea cucumber polysaccharide in our previous study, and both sea cucumber polysaccharide and fermented sea cucumber polysaccharide were sulfated polysaccharides mainly containing fucose. Although no significant differences were observed in the content of total sugar, protein, and crude fat between SC and FSC, the large molecular proteins in the body wall of SC were degraded into small polypeptides after fermentation. The contents of both essential and nonessential amino acids were increased in FSC. This may be related to the natto kinase of *Bacillus natto* [15], which has been proven to effectively degrade sea cucumber protein into peptides and amino acids [13]. Sea cucumber peptides are not only easy to absorb but also important active substances in sea cucumbers [12]. Studies have shown that sea cucumber peptides alleviate acute alcoholic liver injury in mice by enhancing antioxidant responses [16]. Amino acid analysis revealed differences in amino acid composition between FC and FSC, while metabolome analysis revealed differences in products of cellular amino acid metabolism (i.e., creatine, nitric oxide, polyamines, and glutathione) [17], which are vital to the growth, development, health, and survival of humans and other animals [18]. Analyses based on nontargeted metabolomics also showed differences in amino acid metabolism in SC and FSC. The upregulated metabolites related to amino acid metabolism were much more abundant than the downregulated metabolites. It has been reported that the degradation of soybean protein by *Bacillus natto* promotes the diversity of amino compounds [19], which is in agreement with our results. After fermentation, the basic nutrients of SC were preserved, and FSC is more easily absorbed by the human body.

Changes in composition may lead to changes in function. This study examined the efficacy of SC and FSC to alleviate AAI. Ethanol is oxidized to acetaldehyde by ethanol dehydrogenase and then converted into acetic acid by acetaldehyde dehydrogenase, a process that generates hepatic cytochrome P450 2E1 and reactive oxygen species, leading to increased activity of the microsomal ethanol oxidizing system and aggravation of liver injury [20]. Singh and Sharma [21] found that the methanolic extract of Moringa oleifera leaves exhibited antioxidant activity and prevented early alcoholic liver injury by correcting oxidative stress. Hydrogen is an effective antioxidant for various diseases in animals and humans, and Liu et al. [22] showed that inhaled hydrogen ameliorated alcohol-induced liver injury in a mouse model and attenuated hepatic oxidative stress, inflammation, and steatosis. Therefore, administering a novel functional food with high antioxidant capacity is important to intervene in liver injury caused by AAI. Based on the results of the in vitro antioxidant activity of FSC, we investigated the effects and mechanisms of FSC on AAI.

The righting reflex determines how quickly a mouse can return to its normal position after it falls backward with hands and legs in the air. However, when the mice consumed a large amount of ethanol in a short period of time, the high concentration of ethanol in the blood paralyzed the central nervous system, ultimately leading to loss of the righting reflex. First, in behavioral studies, FSC increased the latency and shortened the duration. Although FSC did not change the incidence of loss of the righting reflex in mice, it elevated the tolerance of mice to AAI. In general, the blood alcohol content is determined by the rates of ethanol absorption and metabolism, and increasing the gastric contents could slow the absorption of ethanol. The results indicated that the decrease in blood alcohol content in the FSC group was not caused by slowed ethanol absorption.

When liver injury occurs, the membrane permeability of liver cells increases. Transaminases enter the bloodstream, leading to an increase in plasma transaminase activity. Therefore, plasma transaminase activity is an important indicator of early liver injury, and usually, high aspartate aminotransferase and alanine aminotransferase activities in plasma suggest that liver injury has occurred [23]. SC and FSC significantly reduced the activities of aspartate aminotransferase and alanine aminotransferase in plasma and slowed the hepatocellular injury caused by short-term continuous alcohol intake. The significant reduction in alanine aminotransferase and aspartate aminotransferase activities may be related to their ability to scavenge the highly reactive hydroxyl radicals that result from the metabolism of alcohol to neutralize the oxidative damage to hepatocytes and reduce inflammation [23]. Ethanol dehydrogenase and acetaldehyde dehydrogenase are the key enzymes in the ethanol metabolism pathway, and their activities determine the rate of ethanol clearance in the liver. Both ethanol dehydrogenase and acetaldehyde dehydrogenase play important roles in alcohol metabolism and can reduce the blood alcohol content. FSC had good effects on ethanol dehydrogenase and acetaldehyde dehydrogenase activation, reduced the blood alcohol content and accelerated alcohol metabolism in mice. This suggests that alcohol intake stimulates ethanol dehydrogenase and acetaldehyde dehydrogenase activity in the blood and accelerates intracellular alcohol and acetaldehyde catabolism [24]. Malondialdehyde is one of the end products of hepatic lipid peroxidation, and it reflects the rate and intensity of lipid peroxidation. Moreover, superoxide dismutase is necessary to scavenge oxygen radicals and maintain cellular redox balance. In general, malondialdehyde and superoxide dismutase levels are the main parameters affecting the state of oxidative stress [25]. The elevated malondialdehyde levels and decreased superoxide dismutase activity in the model group mice demonstrated that alcohol intake induced significant oxidative stress. In contrast, both SC and FSC significantly reduced the malondialdehyde content and increased the superoxide dismutase activity in the liver, but FSC produced better effects. In summary, FSC ameliorated the oxidative stress injury in the liver induced by acute alcohol intake. The triglyceride content is a sensitive indicator of lipid metabolism because hepatic steatosis or disorders of hepatic lipid metabolism lead to an increase in triglyceride levels, so triglyceride accumulation leads to the formation of fatty liver. However, the present study found that FSC intake did not alleviate the accumulation of triglycerides in the liver. The effect of FSC on AAI was thus practically confirmed, and so the potential mechanisms were explored. The results indicated that FSC alleviated AAI by improving the activities of alcohol metabolism and antioxidant enzymes in the liver but did not alleviate the accumulation of triglycerides.

After fermentation, in addition to the changes in the main nutrients, there are also great differences in the small molecule metabolites, and many of the active components may affect the function of fermented sea cucumber products. The differentially abundant metabolites were enriched in the arachidonic acid metabolism pathway as determined from both positive and negative ionization modes. The metabolite that was upregulated in the arachidonic acid metabolic pathway in positive ionization mode was prostaglandin E2, one of the major proinflammatory prostaglandins obtained from arachidonic acid via catalysis by the enzyme cyclooxygenase [26]. Prostaglandins are major mediators of inflammation, which plays a central role in the development of heart failure, and studies have shown that prostaglandin E2 can treat and prevent heart failure [27]. In negative ionization mode, the upregulated metabolites involved in arachidonic acid metabolism were leukotriene C4, prostaglandin A2 and prostaglandin G2. Leukotriene C4 is one of the main components of anaphylactic reactions and can cause strong smooth muscle contractions, which ultimately has an antitumor effect [28]. Prostaglandin A2, an endogenous substance derived from arachidonic acid, has antitumor activity and plays a crucial role in the induction of apoptosis [29]. The enriched metabolic pathways and the differentially abundant metabolites may play an important role in the active changes in sea cucumber fermented by *Bacillus natto*. However, due to the large amount of data generated by metabolomics, understanding the functions of the differentially abundant metabolites through only enrichment analysis of metabolic pathways is not sufficient. More metabolites that may affect the quality of fermented sea cucumber products need to be further analyzed and verified in the future.

## 4. Materials and Methods

### 4.1. Materials

*A. japonicus* was purchased from Dalian Xinyulong Marine Biological Seed Technology Co., Ltd. (Dalian, China). *Bacillus natto LY-7* was isolated from the fermented food natto in our laboratory and deposited in the China General Microbiological Culture Collection Center (Beijing, China). Luria–Bertani medium was purchased from Guangdong Huankai Microbial Technology Co., Ltd. (Guangzhou, China). SDS-PAGE precast gels were purchased from Shanghai Wansheng Haotian Biotechnology Co., Ltd. (Shanghai, China). RU21 initial-alcoholic tablets were purchased from American Spirit Sciences Co., Ltd. (Los Angeles, CA, USA). Aspartate aminotransferase, Alanine aminotransferase, Triglyceride, Acetaldehyde dehydrogenase, Ethanol dehydrogenase, Malondialdehyde, Superoxide dismutase, and a bicinchoninic acid protein assay kit were purchased from Beijing Solabo Technology Co., Ltd. (Beijing, China). All other reagents were purchased from Tianjin Damao Reagent Factory (Tianjin, China).

### 4.2. Preparation of Fermented Sea Cucumber

The fresh sea cucumber was gutted, and the body wall was cleaned and soaked in 4 volumes of water for 3 h for desalination. The water was changed every hour. Next, the desalinated body wall was lyophilized and crushed to obtain SC. For fermentation, the medium contained 3.5 g/L sea cucumber powder and 7.5 g/L glucose, and the inoculation amount of *Bacillus natto* seed liquid was 4%. Fermentation was carried out at 35 °C for 48 h. FSC was obtained by lyophilizing the broth.

### 4.3. Determination of the Essential Components

The essential components of both FSC and SC were examined by the direct drying method for the determination of moisture, the phenol sulfate method for the determination of polysaccharides, the Kjeldahl nitrogen method for the determination of proteins, the Soxhlet extraction method for the determination of crude fats, and the high-temperature scorch weight method for the determination of ash. All of these methods were described in a previous work [10].

### 4.4. Protein Characterization

The content of water-soluble protein was determined according to the Biuret method described in Setyani’s work [30]. The molecular mass distribution of water-soluble protein was determined using Tricine-SDS-PAGE. The sample was mixed with sample buffer at a ratio of 1:5. The mixture was boiled for 5 min and a sample volume of 10 μL was applied. Electrophoresis was carried out at a constant 90 V for 30 min, followed by an increase to 120 V, and the whole process was carried out for 2–3 h. After electrophoresis was completed, the gel plate was fixed in Coomassie brilliant blue staining reagent for 1 h, removed from this solution, and placed in decolorizing solution overnight for decolorization until the bands were clear [31].

### 4.5. Amino Acid Composition

Free amino acids were determined by high-performance liquid chromatography methods with *O*-phthalaldehyde precolumn derivatization. Two hundred microliters of the sample mixed with 100 μL of *O*-phthalaldehyde derivative solution for 1 min of reaction, and then 200 μL of KH_2_PO_4_ solution was added to the reaction system. Twenty microliters of the reaction mixture was analyzed by high-performance liquid chromatography. The chromatographic conditions were described in previous studies [32].

### 4.6. Animal Experiments

#### 4.6.1. Animal Feeding Plan

Animal experiments were conducted in accordance with Chinese National Standards: Experimental Animals-Guidelines for Ethical Management of Animal Welfare and were approved by the Animal Ethics Committee of Shenyang Grenster Biotechnology Co., Ltd. (Shenyang, China). The approval number was CSE202303002.

In this experiment, 72 four-week-old SPF C57BL/6 mice were purchased from Changsheng Biotechnology Co., Ltd. (Shenyang, China). The mice were housed in an SPF-grade animal house kept at a temperature of 23 ± 2 °C and relative humidity of 30–40% with alternating 12 h periods of light and dark.

#### 4.6.2. Design of the Animal Experiments

After 7 days of acclimatization, the 72 mice were randomly divided into 6 groups (12 mice in each group) as follows: (1) control group without alcohol or treatment; (2) model group without treatment (gavaged with 0.17 mL/kg body weight ethanol); (3) positive group (gavaged with 300 mg/kg body weight RU21 and 0.17 mL/kg body weight ethanol); (4) SC group (gavaged with 300 mg/kg body weight SC and 0.17 mL/kg body weight ethanol); (5) low dose FSC group (L-FSC, gavaged with 300 mg/kg body weight FSC and 0.17 mL/kg body weight ethanol); and (6) high dose FSC group (H-FSC, gavaged with 600 mg/kg body weight FSC and 0.17 mL/kg body weight ethanol). RU21 contains succinic acid, fumaric acid, alpha-fatty acids, and glutamic acid, as well as cysteine and B-vitamin complexes, which keep the Krebs cycle working and break down more alcohol by supplementing with these acids. Mice were given 300 mg/kg body weight SC, 300 mg/kg body weight FSC, or 600 mg/kg body weight FSC by gavage 30 min prior to alcohol gavage in the SC group, L-FSC, and H-FSC group, respectively. A dose of 300 mg/kg body weight RU21 was administered to mice 30 min prior to alcohol gavage in the RU21 group, while the same volume of saline was administered to the mice in the control group and model group in the same manner. Ethanol (0.17 mL/kg body weight) was then administered to the mice every 24 h for 3 consecutive days to induce the AAI model, except for the mice in the control group, which were given the same volume of saline instead [3].

#### 4.6.3. Behavioral Observations

Immediately after the last gavage, the mice were subjected to behavioral observations. The mice were placed on all fours facing upwards, and if the mice remained in this position for more than 30 s, they were defined as having loss of righting reflex, that is, drunk, but if the righting reflex recovered, they were considered to have recovered. The period from the gavage of alcohol to the disappearance of this reflex was defined as the latency time, and the period from the disappearance of this reflex to its recovery was defined as the duration time. The latency time and duration time were recorded.

#### 4.6.4. Blood Alcohol Content Analysis

Three hours after the last gavage, whole blood was collected from the tailbone. The blood alcohol content in whole blood was measured by headspace gas chromatography according to Goto’s research [33].

#### 4.6.5. Determination of Liver Tissue Biochemical Indices

After whole blood was collected from the tailbone, the mice were sacrificed by anesthesia, and the livers were taken and weighed. The liver index was determined by the ratio of the weight of liver to the weight of the mice. Some of the liver tissues were mixed with 1.0 mL of precooled saline, thoroughly ground, and centrifuged at 4 °C for 10 min. The supernatant was used to determine the activities of ethanol dehydrogenase, acetaldehyde dehydrogenase, and superoxide dismutase and the concentrations of malondialdehyde, triglyceride, and protein. Enzymatic activities were determined by a commercial assay kit according to the manufacturer’s instructions (Beijing Solabo Technology Co., Ltd., Beijing, China).

#### 4.6.6. Determination of Plasma Transaminase

Whole blood was centrifuged, and the plasma was used for determining the activities of aspartate aminotransferase and alanine aminotransferase with commercial assay kits according to the manufacturer’s instructions (Beijing Solabo Technology Co., Ltd., Beijing, China).

### 4.7. Nontargeted Metabolomics Analysis

#### 4.7.1. Metabolite Extraction

The sample was ground with liquid nitrogen, and 100 mg of the ground powder was mixed with 500 μL of 80% methanol solution. The mixture was kept on ice for 10 min and then at 4 °C for 20 min. Next, 100 μL of the supernatant was diluted with 150 μL of water for LC-MS/MS analysis. Six replicates were prepared for each sample, and an equal volume of each sample was mixed to prepare quality control samples.

#### 4.7.2. LC-MS/MS Analysis

Metabolite analyses were carried out on a Vanquish UHPLC system (Thermo Fisher Scientific, Waltham, MA, USA) coupled in tandem to a Q Exactive™ HF-X mass spectrometer (Thermo Fisher Scientific, Waltham, MA, USA) at Novogene Co., Ltd. (Beijing, China). Chromatographic separation was performed on a Hypersil Gold column (2.1 × 100 mm, 1.9 μm) at 40 °C. The injection volume was 1 μL, and the flow rate was 0.2 mL/min. Eluent A for positive and negative ionization modes was 0.1% formic acid solution and 5 mol/L ammonium acetate solution, respectively. Eluent B for positive and negative ionization modes was methanol. The solvent gradient was set as follows: 2% B, 1.5 min; 2–100% B, 3 min; 100% B, 10 min; 100–2% B, 10.1 min; and 2% B, 12 min.

Mass spectrometry analysis was performed in positive/negative ionization mode with a spray voltage of 3.5 kV, capillary temperature of 320 °C, sheath gas flow rate of 35 psi, aux gas flow rate of 10 L/min, S-lens RF level of 60, and aux gas heater temperature of 350 °C.

#### 4.7.3. Data Processing

The mass spectrometry data were processed using Compound Discoverer 3.1 library search software (Thermo Fisher Scientific, Waltham, MA, USA). The retention time deviation was set to 0.2 min, the mass deviation was set to 5 ppm, and the retention time and mass-to-charge ratio of each metabolite were used for peak alignment. The mass bias was 5 ppm, the signal intensity deviation was 30%, and the signal-to-noise ratio was 3. The peak area for each metabolite was quantified. The molecular ion peaks and fragment ions were used to predict the molecular formulae and compared with the mzCloud (https://www.mzcloud.org/, accessed on 1 March 2023), mzVault, and MassList databases; moreover, the raw quantitative results were normalized. Principal component analysis and KEGG (https://www.kegg.jp/, accessed on 2 March 2023) enrichment analysis were completed by Novogene Co., Ltd. (Beijing, China).

### 4.8. Statistical Analysis

Besides LC-MS/MS analysis, each sample was analyzed in triplicate. The data are expressed as the average ± SD. Variance analysis and T tests were used to determine the significance of the differences between the data, and a value of *p <* 0.05 was considered to indicate statistical significance.

## 5. Conclusions

The aim of this study was to understand the changes in the nutrient composition of FSC and their functions in alleviating AAI. The results showed no significant differences in the basic components of SC and FSC. The large molecular proteins of SC were degraded after fermentation, and the amino acid content of FSC was significantly increased. The amino acid metabolites were also increased in FSC as determined by both positive and negative ionization modes, and arachidonic acid metabolism was promoted. FSC increased the latency and shortened the duration of the loss of the righting reflex and significantly decreased the blood alcohol content levels and plasma aspartate aminotransferase and alanine aminotransferase activities in mice with AAI. In addition, FSC significantly reduced the malondialdehyde levels and increased the superoxide dismutase activity in mouse livers. FSC had good effects on ethanol dehydrogenase and acetaldehyde dehydrogenase activation but did not alleviate the accumulation of triglycerides, information that will be beneficial for the development of new sea cucumber fermentation products and the prevention of AAI.

## Figures and Tables

**Table 1:** The basic composition analysis of SC and FSC.

Main Components	SC	FSC
Protein (%)	52.97 ± 1.02 ^a^	56.28 ± 1.12 ^a^
Total sugar (%)	9.17 ± 0.87 ^a^	10.32 ± 0.56 ^a^
Crude fats (%)	2.50 ± 0.12 ^a^	2.60 ± 0.24 ^a^
Ash content (%)	17.38 ± 1.04 ^a^	18.05 ± 0.85 ^a^
Water content (%)	3.97 ± 0.25 ^a^	4.61 ± 0.23 ^a^
Amino acid composition (g/100 g)		
Alanine	1.95 ± 0.02 ^a^	2.67 ± 0.11 ^a^
Arginine	1.71 ± 0.05 ^b^	2.93 ± 0.17 ^a^
Aspartic acid	3.43 ± 0.13 ^a^	3.73 ± 0.22 ^a^
Cysteine	0.18 ± 0.03 ^a^	0.24 ± 0.03 ^a^
Glutamic acid	4.45 ± 0.10 ^a^	6.13 ± 0.25 ^a^
Glycine	4.64 ± 0.03 ^b^	5.28 ± 0.19 ^a^
Histidine	0.72 ± 0.03 ^a^	0.60 ± 0.10 ^b^
Proline	2.94 ± 0.16 ^a^	2.92 ± 0.03 ^a^
Tyrosine	0.77 ± 0.03 ^b^	1.20 ± 0.12 ^a^
Serine	1.45 ± 0.02 ^b^	1.91 ± 0.09 ^a^
Non-essential amino acid	22.24 ^a^	27.61 ^b^
Lysine	2.00 ± 0.05 ^a^	1.86 ± 0.08 ^a^
Isoleucine	1.20 ± 0.08 ^b^	1.44 ± 0.24 ^a^
Methionine	0.70 ± 0.10 ^a^	0.72 ± 0.11 ^a^
Threonine	1.40 ± 0.02 ^b^	1.93 ± 0.10 ^a^
Valine	1.45 ± 0.07 ^a^	1.71 ± 0.10 ^a^
Leucine	1.67 ± 0.06 ^b^	2.01 ± 0.21 ^a^
Phenylalanine	2.10 ± 0.28 ^a^	2.17 ± 0.08 ^a^
Essential amino acid	10.52 ^a^	11.84 ^b^

^ab^ Data having different letters indicates that the difference is significant (*p <* 0.05). SC: sea cucumber; FSC: fermented sea cucumber.

**Table 2:** Effect of FSC on blood alcohol content and behavior.

Groups	Latency Time (min)	Duration Time (min)	Blood Alcohol Content (μg/mL)
Control group	-	-	-
Model group	13.11 ± 3.85 ^a^	239.00 ± 74.56 ^a^	19.33 ± 2.22 ^a^
Positive group	28.07 ± 5.15 ^b^	149.91 ± 5.38 ^b^	13.80 ± 1.51 ^b^
SC group	14.58 ± 5.05 ^a^	237.25 ± 48.04 ^a^	15.37 ± 3.78 ^a^
L-FSC group	16.01 ± 3.71 ^a^	208.44 ± 58.59 ^a^	14.13 ± 1.93 ^b^
H-FSC group	23.61 ± 6.67 ^b^	169.11 ± 18.18 ^b^	13.53 ± 2.39 ^b^

^ab^ Data having different letters indicates that the difference is significant (*p* < 0.05). -: not detected.

**Table 3:** Effect of FSC on liver injury.

Groups	Liver Index (%)	TG (mmol/g)	ALT (U/mL)	AST (U/mL)
Control group	4.46 ± 0.87 ^a^	0.049 ± 0.012 ^a^	0.699 ± 0.227 ^a^	2.625 ± 0.152 ^a^
Model group	4.97 ± 0.45 ^a^	0.087 ± 0.029 ^b^	1.600 ± 0.435 ^b^	3.918 ± 0.357 ^b^
Positive group	4.68 ± 0.37 ^a^	0.066 ± 0.010 ^b^	1.392 ± 0.337 ^b^	4.059 ± 0.450 ^b^
SC group	4.90 ± 0.29 ^a^	0.072 ± 0.017 ^b^	0.901 ± 0.132 ^a^	2.692 ± 0.127 ^a^
L-FSC group	4.84 ± 0.41 ^a^	0.076 ± 0.024 ^b^	0.621 ± 0.267 ^a^	2.713 ± 0.126 ^a^
H-FSC group	4.70 ± 0.46 ^a^	0.069 ± 0.008 ^b^	0.899 ± 0.209 ^a^	2.562 ± 0.238 ^a^

^ab^ Data having different letters indicates that the difference is significant (*p* < 0.05). TG: triglyceride; ALT: alanine aminotransferase; AST: aspartate aminotransferase; SC: sea cucumber; L-FSC: low dose fermented sea cucumber; H-FSC: high dose fermented sea cucumber.

## Data Availability

Data is contained within the article or Supplementary Material.

## Supplementary Materials

The following supporting information can be downloaded at: https://www.mdpi.com/article/10.3390/foods13020262/s1, Table S1: Amino acid differential metabolites of FSC and SC (negative ionization mode); Table S2: Amino acid differential metabolites of FSC and SC (negative ionization mode). Raw data: Total ion flow chart and screened differential metabolites.

## Abbreviations

AAI	Acute alcohol intoxication
SC	Sea cucumber
FSC	Fermented sea cucumber
KEGG	Kyoto Encyclopedia of Genes and Genomes
SC group	Sea cucumber group
L-FSC group	Low dose fermented sea cucumber group
H-FSC group	High dose fermented sea cucumber group
