# Supplementary material for: Patterns of the Nutrients and Metabolites in Apostichopus japonicus Fermented by Bacillus natto and Their Ability to Alleviate Acute Alcohol Intoxication
Source: Foods. 2024 Jan 14;13(2):262. doi: 10.3390/foods13020262 (PMC10814447; doi:10.3390/foods13020262)
Supplement: Supplementary file 1 [file foods-13-00262-s001.zip › foods-2718425-supplementary/supplement/foods-2718425-supplementary.pdf]

Table S1. Amino acid differential metabolites of FSC and SC (negative ionization mode)

Metabolic pathway	Up-regulated differential metabolites	Down-regulated differential metabolites
tyrosine metabolism (Tyr)	Tyrosine, norepinephrine, epinephrine, tyramine, 3-methoxytyramine	3,5-Diiodo-L-tyrosine, 3-Iodo-L-tyrosine, Gallic acid
Metabolism of alanine, aspartate and glutamate	L-Arginine succinate	α-Ketoglutaric acid
tryptophan metabolism (Trp), an essential amino acid	Melatonin, indole	Indole 3acetamide; Kynurenine; D-Kynurenine
Glycine, serine and threonine metabolism	L-Threonine, Cystathionine	betaine
Cysteine and methionine metabolism	Methionine, cystathionine	glutathione

Table S2. Amino acid differential metabolites of FSC and SC (negative ionization mode)

metabolic pathway	Up-regulated differential metabolites	Down-regulated differential metabolites
tyrosine metabolism (Tyr)	Indole-5,6-quinone, 3,4-dihydroxy-L-phenylalanine, 4-hydroxyphenethyl alcohol, homovanillic acid, hydroquinone	fumaric acid
Histidine metabolism	L-Aspartic acid, imidazoleacetic acid	L-glutamic acid, 1-methylhistamine
Phenylalanine metabolism	N-acetyl-L-phenylalanine, D-phenylalanine, phenylpyruvic acid, trans-cinnamic acid	fumaric acid
Arginine and proline metabolism	Ethyl 5-aminopentanoate, 4-oxo-L-proline	L-Glutamic acid
Valine, leucine and isoleucine biosynthesis	2-Isopropylmalic acid	-
Lysine biosynthesis	L-Saccharin	L-Aspartic acid

-: not detected.
